# Oxytocin Disturbs Vestibular Compensation and Modifies Behavioral Strategies in a Rodent Model of Acute Vestibulopathy

**DOI:** 10.3390/ijms232315262

**Published:** 2022-12-03

**Authors:** Guillaume Rastoldo, Emna Marouane, Nada El-Mahmoudi, David Péricat, Brahim Tighilet

**Affiliations:** 1 Laboratoire de Neurosciences Cognitives (LNC), UMR 7291, Aix Marseille Université-CNRS, 13331 Marseille, France; 2 UNICAEN, INSERM, COMETE, CYCERON, CHU Caen, Normandie Université, 14000 Caen, France; 3 Institut de Pharmacologie et de Biologie Structurale (IPBS), Université de Toulouse, CNRS, 31000 Toulouse, France

**Keywords:** vestibular compensation, oxytocin, hormone, behavioral study

## Abstract

Unilateral inner ear injury is followed by behavioral recovery due to central vestibular compensation. The therapeutic effect of oxytocin (OT) on vestibular compensation was investigated by behavioral testing in a rat model of unilateral vestibular neurectomy (UVN). Animals in the oxytocin group (UVN-OT) exhibited delayed vestibular compensation on the qualitative scale of vestibular deficits and aggravated static postural deficits (bearing surface) compared to animals in the NaCl group (UVN-NaCl). Surprisingly, oxytocin-treated animals adopt a different postural strategy than untreated animals. Instead of shifting their weight to the ipsilesional paws (left front and hind paws), they shift their weight to the front paws (right and left) without modification along the lateral axis. Furthermore, some locomotor strategies of the animals to compensate for the vestibular loss are also altered by oxytocin treatment. UVN-OT animals do not induce an increase in the distance traveled, their mean velocity is lower than that in the control group, and the ipsilesional body rotations do not increase from 7 to 30 days after UVN. This study reveals that oxytocin treatment hinders the restoration of some postural and locomotor deficits while improving others following vestibular lesions. The mechanisms of the action of oxytocin that support these behavioral changes remain to be elucidated.

## 1. Introduction

Unilateral vestibular loss causes vestibular syndrome in most species, including humans. This syndrome is characterized by a cascade of functional disorders, including postural imbalance, altered gait, spontaneous nystagmus, altered vestibular eye reflexes, and cognitive and neurovegetative disorders. The various symptoms that constitute the vestibular syndrome decline progressively, each with its kinetics, through the phenomenon of “vestibular compensation” [1,2]. The restoration of the homeostatic excitability level between the homologous vestibular nuclei is closely related to the restoration of vestibular functions [2,3,4].

Different strategies are implemented in the deafferented vestibular environment to restore the level of homeostatic excitability, which is crucial for functional recovery. One of these strategies is to reduce the expression of KCC2 co-transporters, thus, promoting a depolarizing action of GABA during the first days after unilateral vestibular neurectomy (UVN) [5,6,7].

A recent study indicates that oxytocin and its receptor enable the upregulation, phosphorylation, insertion, and stabilization of the KCC2 co-transporter at the membrane [8]. Oxytocin released massively during delivery acts as a neuroprotective agent by inhibiting fetal neurons to increase their resistance to anoxia [9,10]. Indeed, in immature neurons, GABA is excitatory due to the low expression of KCC2 and, consequently, a high concentration of intracellular chloride (Cl^−^) ions [11,12,13]. Depolarizing GABA during the fetal and postnatal periods helps promote the excitatory synaptic inputs necessary for brain plasticity and development [10,11,14,15]. We have recently hypothesized in our UVN animal model the re-emergence of a critical period during the early post-lesion days that are reminiscent of certain developmental stages, during which neurogliogenesis, BDNF, and KCC2 downregulation are engaged in ipsilesional vestibular nuclei [5,7]. Thus, the oxytocin-induced upregulation of KCC2 could disrupt plasticity mechanisms during this critical period and delay vestibular compensation. The activation of oxytocin receptors also stimulates the local synthesis of allopregnanolone (an allosteric modulator of GABA_A_ receptors), which in turn increases the GABA-mediated inhibitory tone [16]. It is then possible that oxytocin potentiates GABAergic inhibition in the vestibular nuclei due to the presence of GABA_A_ and OT receptors in these structures [17,18,19]. Oxytocin may, therefore, also impede the kinetics of vestibular compensation by targeting the GABA_A_ receptor [5,17].

In the present study, we aimed to investigate the behavioral consequences of acute pharmacological treatment with oxytocin in adult rats subjected to unilateral vestibular neurectomy. Using a behavioral approach, we quantified the impact of this treatment on vestibular syndrome and the recovery of vestibular functions.

## 2. Results

### 2.1. Overall Assessment

**Figure 1:**
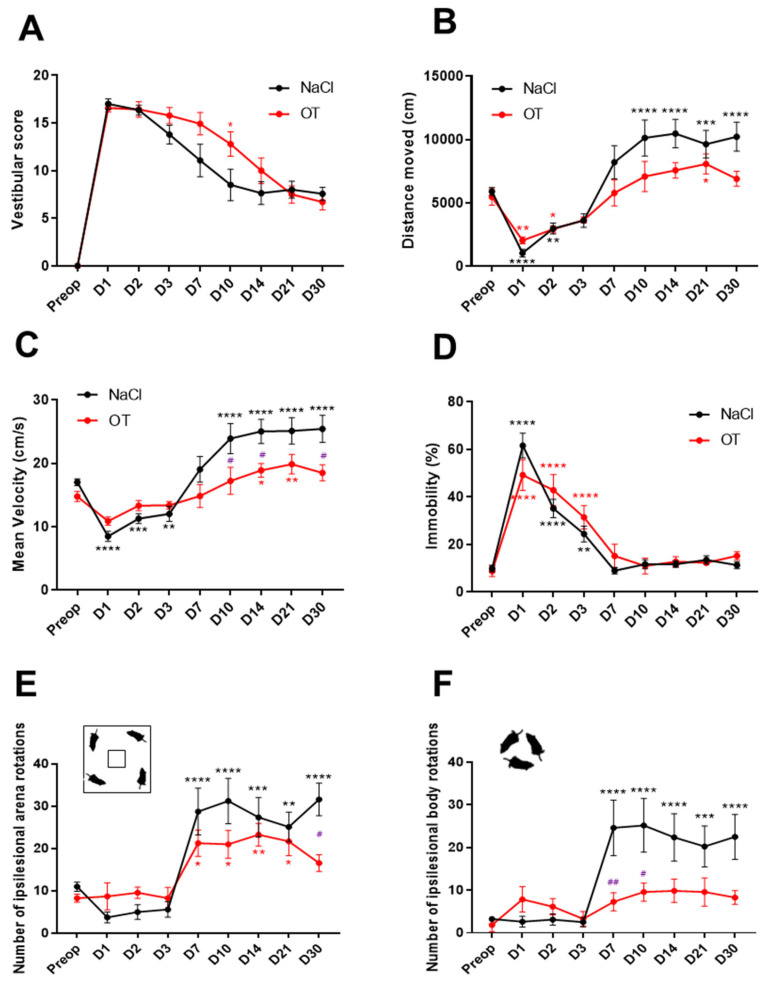
Behavioral assessment of the vestibular syndrome of oxytocin-treated animals. (**A**) Qualitative assessment of the vestibular syndrome over postlesion days of control UVN rats (NaCl in black, *n* = 8) and oxytocin-treated UVN rats (OT in red, *n* = 7). (**B**) Assessment of the distance traveled by UVN-NaCl and UVN-OT rats in the open field. (**C**) Assessment of the mean velocity by UVN-NaCl and UVN-OT rats in the open-field. (**D**) Assessment of the % of time immobile by UVN-NaCl and UVN-OT rats in the open-field. (**E**) Mean number of ipsilesional (left) arena rotations by UVN-NaCl and UVN-OT rats in the open-field. (**F**) Mean number of ipsilesional body rotations by UVN-NaCl and UVN-OT rats in the open-field. Error bars represent standard error of the mean (SEM). * *p* < 0.05, ** *p* < 0.01, *** *p* < 0.001, **** *p* < 0.0001. A significant difference from the preoperative value is indicated by * in black for the UVN-NaCl group and in red for the UVN-OT group. A significant difference between the UVN-NaCl and UVN-OT rats is indicated by # in purple.

After UVN, the animals showed posturo-locomotor alteration characteristics of the vestibular syndrome. These behavioral deficits were assessed using a qualitative “vestibular score” scale (presented in the Materials and Methods Section). In both groups, the mean vestibular score peaked on day 1 post-lesion (NaCl: 17 ± 0.39; OT: 16.57 ± 0.53) and reached a plateau of around D10 for the UVN-NaCl and D21 for UVN-OT group (Figure 1A). At this stage, vestibular deficits never completely disappeared, and several residual impairments were found, such as head tilt, tail-hanging reflex, and locomotion impairment. When comparing the two groups, UVN-OT-treated animals have a significantly higher vestibular score at D7 post-lesion compared with UVN-NaCl animals (*p* < 0.05), illustrating a delay in the vestibular compensation of these animals.

Several locomotor parameters were analyzed during 10 min of free exploration in an 80 *×* 80 cm open field. As recently demonstrated [20], UVN-NaCl rats show a significant decrease in the distance traveled the first days after the lesion (D1: *p* < 0.0001; Day 2: *p* < 0.01). From D10 to D30, the mean distance traveled by UVN-NaCl rats increased significantly compared to before UVN (D10, 14 and 30: *p* < 0.0001; D21: *p* < 0.001). In comparison, OT-treated animals decreased their distance traveled only on the first two days after injury (D1: *p* < 0.01; D2: *p* < 0.05), but there was no increase in the subsequent delays except at D21 (*p* < 0.05 Figure 1B). No significant differences were observed between the two groups at the different post-lesion time points.

The mean velocity of UVN-NaCl rats followed the same kinetics as the distance traveled: a significant decrease on the first 3 days post-UVN (D1: *p* < 0.0001; D2: *p* < 0.001; D3: *p* < 0.01) followed by a significant increase from D10 to D30 (D10 to D30: *p* < 0.0001) compared to preoperative values (Figure 1C). Conversely, OT-treated animals did not significantly decrease their mean velocity during the first 3 days post-UVN, but a significant increase was found at D14 and D21 (D14: *p* < 0.05; D21: *p* < 0.01). However, the mean velocity of UVN-OT animals is significantly lower at D10, D14, and D30 compared to UVN-NaCl animals (NaCl vs. OT: *p* < 0.05).

The percentage of immobility time for rats after UVN reflects the inability of the animal to move properly after the lesion and correlates with the decrease in the distance traveled, which was observed in the first few days post-UVN. The percentage of immobility time for rats in the UVN-NaCl and UVN-OT groups increased significantly on the first 3 days after the lesion with a maximum value on day 1 (NaCl: D1 and D2: *p* < 0.0001; D3: *p* < 0.01; OT: D1 to D3: *p* < 0.0001) and then returned to preoperative values from D7 to D30 (Figure 1D).

We highlighted two locomotor behaviors of unilaterally neurectomized animals in our previous paper [20]: an increase in ipsilesional (left) body rotations (associated with circling behavior) and an increase in ipsilesional rotations of the animals in the arena (associated with a preference to explore the open field in the direction of the lesion). Both of these behaviors were also assessed for OT-treated animals. The kinetics of the number of ipsilesional rotations associated with circling (Figure 1E) or in the arena (Figure 1F) of UVN-NaCl animals followed the same kinetics: no change during the first 3 days post-lesion, then a significant increase from D7 to D30 (Figure 1E: D7, D10, D14, and D30: *p* < 0. 0001; D21: *p* < 0.001; Figure 1F: D7, D10, and D30: *p* < 0.0001; D14: *p* < 0.001; D21: *p* < 0.01). On the contrary, OT-treated animals did not have more circling regardless of the postoperative time (Figure 1E) and differed significantly from the UVN-NaCl group at D7 and D10 (D7: *p* < 0.01; D10: *p* < 0.05). However, the number of ipsilesional rotations in the arena of UVN-OT animals was similar to the UVN-NaCl group: no change during the first 3 days post-lesion and then a significant increase from D7 to D21 (OT: D7, D10, and D21: *p* < 0.05; D14: *p* < 0.01). Surprisingly, the UVN-OT group differed significantly from the UVN-NaCl group at D30 post-lesion (D30: *p* < 0.05).

### 2.2. Weight Distribution

**Figure 2:**
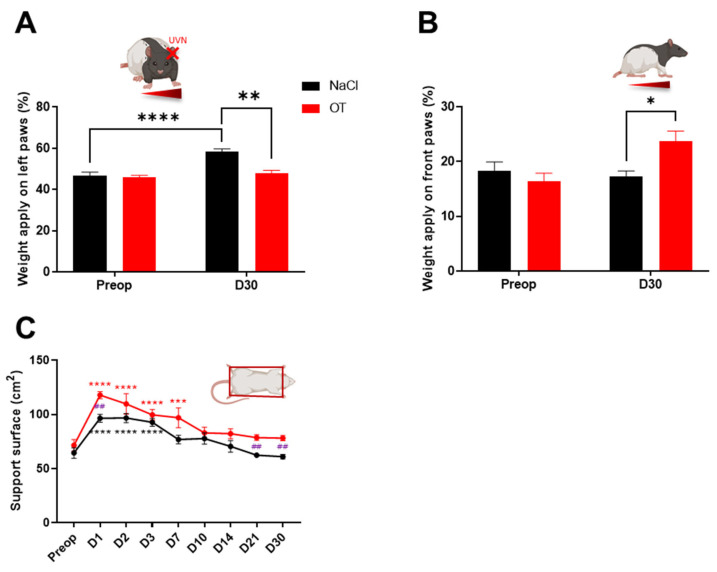
Evaluation of postural and weight distribution of oxytocin-treated animals. (**A**) Assessment of weight distribution on the lateral axis at pre-operative (Preop) time and 30 days post-UVN in UVN-NaCl rats (black ***n***=8) and UVN-OT rats (red *n* = 7). (**B**) Assessment of weight distribution on the anteroposterior axis at pre-operative (Preop) time and 30 days post-UVN in UVN-NaCl and UVN-OT rats. (**C**) Assessment of the support surface area in UVN-NaCl and UVN-OT rats. Error bars represent standard error of the mean (SEM). * *p* < 0.05, ** *p* < 0.01, *** *p* < 0.001, **** *p* < 0.0001. A significant difference from the preoperative value is indicated by * in black for the UVN-NaCl group and in red for the UVN-OT group. A significant difference between the UVN-NaCl and UVN-OT rats is indicated by # in purple.

The weight distribution on the lateral axis of the animals evaluated with the Bioseb DWB device (see Materials and Methods Section) represents a good index of postural stability [21,22]. Rats distribute their weight equally (about 50%) between the left and right paws before UVN (Figure 2A). In contrast, on the anterior-posterior axis, rats in the UVN-NaCl group distributed an average of 17.52% of their weight to the front paws and 73.48% to the rear paws (Figure 2B). At 30 days post-UVN, rats in the UVN-NaCl group significantly distributed their weight on their ipsilesional (left) paws (*p* < 0.0001) compared to the preoperative time (Figure 2A) with no change in weight on the anteroposterior axis (Figure 2B). On the contrary, animals in the UVN-OT group did not change their weight distribution on the lateral axis compared to the preoperative values (NaCl vs. OT D30: *p* < 0.01; Figure 2A) but significantly increased their weight on the front paws (NaCl vs. OT D30: *p* < 0.05; Figure 2B). The UVN-OT animals adopted a different postural strategy than the UVN-NaCl animals, which consisted of not tilting their weight on the lateral axis but increasing the weight applied on the front legs in the anteroposterior axis.

### 2.3. Support Surface

After UVN, NaCl-injected animals significantly increased their support surface area during the first 3 days post-lesion (D1 to D3: *p* < 0.0001) and then returned to preoperative values from D7 onwards (Figure 2C). For the UVN-OT group, the support surface area increased significantly up to 7 days post-UVN (D1 to D3: *p* < 0.0001, D7: *p* < 0.001) and then returned to preoperative values only from D10 onwards. In addition, the support surface area of OT-treated animals was significantly larger at 1, 21, and 30 days post-UVN compared with the control animals (D1, D21, and D30: *p* < 0.01).

## 3. Discussion

We have shown that oxytocin treatment during the first 3 days post-UVN alters the vestibular compensation in rats. In general, the vestibular compensation of OT-treated rats appears to be delayed. The qualitative scale and the surface of the sustentation polygon show a return to preoperative or compensated values from D10 onwards instead of a return between D3 and D7 in untreated rats. However, surprisingly, oxytocin-treated animals adopt a different postural strategy than untreated animals. Instead of shifting their weight to the ipsilesional paws (left front and hind paws), oxytocin-treated animals shift their weight to the front paws (right and left) without modification along the lateral axis. This strategy is, in fact, adopted very early in OT-treated animals. Their weight is significantly distributed on the two front paws from the first to the 30th day, whereas control UVN rats only tilt their weight forward on the first day after UVN and then return to normal values [22]. Furthermore, some locomotor strategies of the animals to compensate for the vestibular loss are also altered by oxytocin treatment. Oxytocin-treated animals do not induce an increase in the distance traveled, their mean velocity is lower than the control group, and ipsilesional body rotations after UVN do not increase from 7 to 30 days after the lesion.

### 3.1. Oxytocin Disturbs Some Locomotor Parameters in Animals Subjected to Unilateral Vestibular Neurectomy

The behavioral effects of oxytocin on UVN rats may be the result of a direct action on the vestibular nuclei (the presence of the oxytocin receptor in vestibular nuclei: [18,19]) or an indirect action on other brain structures. Since dynamic deficits involve different brain structures and neural networks [1], in order to explain the behavioral effect of oxytocin on locomotion, walking velocities, and body rotations, we favor the second hypothesis. Indeed, an injection of oxytocin at 1 mg/kg (i.p., the same dose and route of administration as we used) has been shown to attenuate an increase in locomotor activity induced by cocaine or methamphetamine intake in rats [23,24]. In contrast, the injection of oxytocin into cocaine-naïve mice does not alter spontaneous locomotor activity [25]. Although the mechanisms underlying oxytocin-induced effects are unclear, it appears that the ability of oxytocin to modulate psychostimulant-induced hyperactivity is closely linked to dopaminergic neurotransmission in mesolimbic brain regions [26]. Interestingly, oxytocin also blocks hyperlocomotion after UVN, and it has been reported that unilateral labyrinthectomy causes a bilateral increase in the dopaminergic D1 and D2 receptors in the striatum [27]. In addition, there is considerable evidence in animals to suggest that vestibular information is transmitted to the basal ganglia and striatum in particular [28,29,30,31,32]. One could postulate that the increased locomotion observed in UVN animals is associated with increased dopaminergic neurotransmission. This hypothesis has already been proposed following studies on genetically modified animal models exhibiting spontaneous circling behaviors [33,34,35]. Stiles and Smith further propose that circling is a dopaminergic imbalance in striatal pathways [36]. Furthermore, the administration of the dopaminergic D2 antagonist (raclopride) causes a reduction in locomotor hyperactivity and circling behavior in these animal models [35]. Yet, we also demonstrate that oxytocin-treated UVN rats do not show an increase in ipsilesional body rotations compared to UVN-NaCl animals. This result reinforces the hypothesis of a modulation of dopaminergic neurotransmission by oxytocin via its receptors at the striatal complex, known to be involved in motor function. Thus, oxytocin would modulate dopaminergic neurotransmission, by acting on its receptors at the striatal complex, and thus blocking the hyperlocomotion and ipsilesional body rotations induced by UVN.

Another hypothesis to explain the absence of hyperlocomotion in UVN-OT animals could reside in the similarity between arginine vasopressin (AVP) and oxytocin. Indeed, the hormone oxytocin differs only from AVP by two amino acids [37]. The similar homology between these hormones and the low selectivity of oxytocin could result in the activation of peripheral vasopressin receptors. In light of recent findings by Wolfe et al., the locomotor inhibition induced by OT administration is mediated by the peripheral vasopressin receptor V1aR and not by the oxytocin receptor (OTR) in the brain [38]. Since we do not use the OTR-specific agonist or V1aR antagonist, we cannot support OTR-exclusive results to explain our behavioral findings. However, in the study of Wolfe et al., a decrease in the distance traveled following the subcutaneous administration of oxytocin was observed in the 60 min following the injection [38]. In our study, the absence of hyperlocomotion in the UVN-OT group was observed from the 10th day until 1 month, which is at least 1 week after the last intraperitoneal injection of oxytocin (the last injection on day 3 post-UVN). This 7-day delay between the last oxytocin injection and the alteration in locomotion makes it unclear whether this long-term outcome is still mediated by a peripheral effect of AVP via its V1aR receptor.

### 3.2. Oxytocin Induces a Change in Postural Strategy in Animals Subjected to Unilateral Vestibular Neurectomy

The shifting of weight onto the ipsilesional paws seemed to be a postural strategy implemented by the animals to maintain their balance after the unilateral loss of vestibular inputs [21,22]. However, it appears that with pharmacological treatments beneficial to vestibular compensation [6] or with sensorimotor rehabilitation [39], this strategy can be lifted. Symmetrical lateralization of the weight of the animals seems beneficial for efficient animal posture stability in view of these results. However, the chronic forward weight distribution observed in oxytocin-treated UVN animals appears to be a deleterious consequence of the treatment. The forward weight shift, observed only on the first post-lesion day, is a consequence of the surgery because this strategy is also observed in animals with sham surgery (opening the skin planes up to the tympanic bulla without damage to the vestibular apparatus). For UVN-OT animals, this strategy becomes chronic and persists until D30. Rats treated with L-Thyroxine [6], which greatly improves the vestibular compensation, do not tilt their weight forward beyond the first-day post-UVN. Therefore, we speculate that the increased weight on the front legs observed in the UVN-OT animals is deleterious to the postural stability of the animal. In support of our hypothesis, the support surface, which is a very good index of postural balance, remains significantly higher in the UVN-OT group at D21 and 1 month after UVN compared to the UVN-NaCl group.

### 3.3. Oxytocin Delays Vestibular Compensation?

UVN animals treated with oxytocin show delayed vestibular compensation since the vestibular score reaches a plateau later than control animals, as well as a slower recovery of vestibular deficits. Moreover, concerning the support surface of the UVN-OT group, significantly higher values were observed on the first day but also later at 21 and 30 days after the vestibular lesion. It has been reported that oxytocin receptor activation also stimulates local allopregnanolone synthesis (an allosteric modulator of GABA_A_ receptors) [16]. Since oxytocin and GABA_A_ receptors are present in vestibular nuclei, it is possible that oxytocin potentiates GABAergic inhibition in both ipsi and contralesional vestibular nuclei indirectly via allopregnanolone. An increased ipsilesional GABAergic inhibition would accentuate the electrophysiological imbalance between the homologous vestibular nuclei, causing a delay in the vestibular compensation. It would have been interesting to relate this result to the membrane expression of KCC2 in the vestibular neurons. Indeed, we have shown that the deafferented vestibular neurons reduce the expression of KCC2 co-transporters, which would favor an excitatory depolarizing action of GABA during the first days after unilateral vestibular neurectomy [5]. Oxytocin, on the other hand, plays a crucial role in controlling the GABA developmental polarity shift in pups during labor [8,9,10]. Similar to the UVN model, the nerve injury model also induces the downregulation of KCC2, and recently, in this model, Ba et al. demonstrated in adult mice that the continuous infusion of oxytocin for three days increased KCC2 expression and function [40]. Based on our behavioral results, if oxytocin restored KCC2 expression in the ipsilesional vestibular nuclei, then allopregnanolone would enhance the inhibitory action of GABA, leading to a worsening of the syndrome and delayed vestibular compensation. On the contrary, if KCC2 co-transporters were not upregulated by oxytocin, then GABA would have a rather excitatory effect (or a decrease in its inhibitory efficacy) in the ipsilesional vestibular nuclei which should reduce the electrophysiological asymmetry between the homologous vestibular nuclei and promote vestibular compensation. Therefore, the observed delay in vestibular compensation after oxytocin treatment strongly suggests an up-regulation of the KCC2 membrane expression induced by this hormonal neuropeptide.

However, to temper our hypothesis, oxytocin-treated animals do not show a shift in weight on ipsilesional paws or an increase in ipsilesional body rotations. Weight lateralization involves muscle tone, which is directly related to the vestibulospinal pathways. An electrophysiological imbalance in the vestibular nuclei is reflected in the vestibulo-spinal pathways causing an asymmetry in muscle tone. However, this is not the case for UVN-OT animals, which present a lateralization of their weight. Moreover, the body rotations of animals after vestibular damage also reflect an imbalance in the vestibular network, probably involving the striatum. Again, oxytocin-treated animals show less ipsilesional body rotations suggesting, as for weight lateralization, a return to homeostatic balance within the vestibular nuclei. Thus, can we really speak of a delay in the compensation induced by oxytocin treatment? Some measured parameters show a delay (support surface, qualitative scale of deficits), while others seem to show the opposite (absence of a decrease in velocity the first three days post-injury and absence of an increase in ipsilesional body rotations). In all cases, the animals seem to have adapted differently to the vestibular lesion and have implemented new postural strategies to compensate for their deficits compared to the untreated animals.

## 4. Materials and Methods

### 4.1. Animals

The experiments were performed on 16 adult female Long Evans rats (250–300 g) originating from our own breeding and from parents obtained from Charles River (St Germain sur l’Arbresle, France). All experiments were performed in accordance with the European Union 2010/63/EU Directive and under the veterinary supervision and control of the National Ethical Committee (French Agriculture Ministry Authorization: B13-055-25). The present study was specifically approved by the Neurosciences Ethic Committee N°71 of the French National Committee on animal experimentation. Every attempt was made to minimize both the number and the suffering of animals used in this experiment. The animals were housed in groups of four in a large, confined space with a 12 h light/dark cycle and with food and water available ad libitum.

### 4.2. Unilateral Vestibular Neurectomy

Female rats were subjected to a left-side vestibular nerve section (*n* = 16) following the surgical procedure previously reported in the literature [41]. To ensure analgesic coverage during surgery, subcutaneous injection of buprenorphine (Buprecare^®^; 0.02 mg/kg) was performed before placing the rats in the anesthesia induction box for 5 min (4% isoflurane concentration). Once anesthetized, the animals were intubated, and anesthesia was maintained at a 3% isoflurane concentration for the rest of the surgery. Briefly, the cervical muscle planes were dissected until the tympanic bulla appeared, which was drilled to expose the promontory containing the cochlea. After exposing the cochlear nerve by drilling the cochlea, the cochlear nerve meatus was enlarged, leading to the vestibulocochlear nerve, which was sectioned at its entry into the brainstem after the aspiration of Scarpa’s ganglion. Before awakening, the wound was closed using a stapler, and the animals received either an intraperitoneal injection of 0.9% NaCl (UVN-NaCl group) or a solution of oxytocin (UVN-OT group, 1 mg/kg). A solution of Ringer Lactate (Virbac, Westlake, TX, USA; 10 mL/kg) was also administered subcutaneously in order to alleviate the dehydration resulting from the inability of the animals to drink normally as a result of the injury. The rats were housed in individual cages after the surgery until the end of the behavior tests.

### 4.3. Study Design

Rats were randomly assigned to the following groups:

UVN-NaCl group: the rats were subjected to UVN and saline (0.9%), which was administered (0.3 mL, i.p) at the end of the surgery and one injection/day during the first 3 days post-lesion (*n* = 8).

UVN-OT group: the rats were subjected to UVN, and Oxytocin (Sigma, St Louis, MO, USA, Cat#O3251) was administered (1 mg/kg in 0.3 mL, i.p) at the end of the surgery and one injection/day during the first 3 days post-lesion (*n* = 7).

### 4.4. Criteria for Exclusion

Animals were excluded from the study if the following symptoms were observed:

- A loss of body weight equal to more than 20% of the preoperative value.
- If the facial nerve had been sectioned.
- Abnormalities in behavioral scoring, i.e., the inability of the animal to stand on all four paws after 5 days post-UVN, convulsions, hemiataxia, etc.

Based on these criteria, one animal of the UVN-OT group was excluded.

### 4.5. Qualitative Assessment of the Vestibular Syndrome

The vestibular syndrome was scored after UVN based on 10 behavioral components previously described [6,7,42]: the tail-hanging test, rearing, grooming, displacement, head-tilt, barrel rolling, retropulsion, circling, and bobbing. The tail-hanging test (adapted from [43,44,45]), quantifies typical vestibulo-spinal reflexes.

- Tail hanging behavior: animals were picked up from the ground at the base of the tail, and body rotation was scored from 0 points (no rotation) to 3 points (several rotations of 360°).
- Landing reflex: after the animals were picked up from the ground at the base of the tail, we scored the first 3 landings from 0 (presence of a landing reflex on the 3 landings) to 3 points (absence of landing reflex on the 3 landings). When lifted by the tail, the control rats exhibited a landing reflex consisting of a forelimb extension that allowed them to land successfully (i.e., they landed on all four legs). The rats with impaired vestibular function do not exhibit a forelimb extension, spin or bend ventrally, sometimes “crawling” up toward their tails, causing them to miss their landings.
- Rearing: the ability of the rat to rear was scored from 0 points (rearing is observed) to 1 point (rearing is absent).
- Grooming: the ability of the rat to groom correctly was scored as follows: 0 points (correct grooming of full body), 1 point (grooming of the face, belly, and flanks but not the base of the tail), 2 points (grooming of the face and belly), 3 points (grooming of the face), 4 points (inability of the animal to groom itself).
- Displacement: the quality of the displacement of the rat was scored from 0 (displacement of the rat with no visible deficit) to 3 points (several deficits in the displacement of the rat).
- Head tilt was scored by estimating the angle between the jaw plane and the horizontal from 0 points (absence of a head tilt) to 3 points (for a 90° angle).
- Barrel rolling was scored as follows: 0 points (absence of barrel rolling), 1 point (barrel rolling evoked by an acceleration in the vertical axis of the rat in our hand), and 2 points (spontaneous barrel rolling).
- Retropulsion characterizing backward movements was scored from 0 (absence of retropulsion) to 1 point (presence of retropulsion).
- Circling was scored from 0 points (absence of circling behavior) to 1 point (presence of circling behavior).
- Bobbing was related to rapid head tilts to the side and was scored from 0 points (absence of bobbing) to 1 point (presence of bobbing).

A mean score was calculated by summing the points obtained for the different quantified components. For each rat, a mean score was collected on the day before the lesion as a baseline value and on days (D) 1, 2, 3, 7, 10, 14, 21, and 30 post-lesion.

### 4.6. Body Weight Distribution

To quantify the postural syndrome following UVN, we used a device (DWB1^®^, Bioseb, Vitrolles, France) measuring the weight distribution of the animals. This device has previously been described for the assessment of postural instabilities in the same model of vestibular loss [21,22]. The device consists of a Plexiglas cage (25 × 25 cm) in which the animal is placed for 5 min recording while moving freely. The floor of the cage is fully covered with a plate equipped with 2000 force sensors. The sensors detect vertical pressure at a sample rate of 30 Hertz. They are connected to an electronic interface that converts the current flowing through it into a measure of weight, with the whole being connected to a computer. The cage is closed by a lid on which is attached a high-definition camera, also connected to the computer through a USB cable. We measured the weight distributed on the lateral axis (the two ipsi- vs. contralesional paws) and the anteroposterior axis (the two front paws vs. hind paws). In order to compensate for the variability in rat weight, the weight distribution on the paws was expressed as a percentage of the animal’s body weight on the day of acquisition. The pre-operative session was recorded the day before the surgery, and then, the time course of the vestibular syndrome was studied on days (D) 1, 2, 3, 7, 10, 14, 21, and 30 post-lesion.

### 4.7. Support Surface

The static postural deficits and recovery were evaluated by measuring the support surface delimited by the four legs of the rat. The support surface can be regarded as a good estimate of postural control because it reflects the behavioral adaptation of the rat compensating for the static vestibulospinal deficits induced by the vestibular lesion. To quantify the support surface, the rats were placed in a device with a graduated transparent floor that allowed them to be filmed from underneath. To avoid measures when the animals were moving, we measured the support surface area when the animal landed. For this purpose, we picked up the animal by the tail and lifted it vertically to a height of about 50 cm (lift duration 2 s; position holding at upper position: 1 s) and dropped it to a height of about 10 cm. When the animal landed on the ground, we took a picture of the location of the four paws. Twenty repeated measurements were taken for each rat, which was tested at each time point (pre-lesion, days (D) 1, 2, 3, 7, 10, 14, 21, and 30 post-lesion), and an average was calculated for each experimental session. The support surface was measured using a custom-written image analysis tool (Matlab^®^, Mathworks, Inc., Natick, MA, USA). For each rat, the data recorded after UVN were compared to the reference value obtained in the same animals.

### 4.8. Open Field Test

To quantify the locomotor syndrome following UVN, we used an automated video tracking software (EthoVision™ XT 14, Noldus, The Netherlands). Animals were individually placed in a square open field (80 × 80 × 40 cm) and were allowed to move freely. Their behavior was recorded for 10 min using a digital camera and then analyzed with EthoVision™ XT 14 software. The position of the nose, body center, and tail were automatically detected by video software (for technical details, see [20]). We measured the mean distance traveled, mean locomotor velocity, the mean percentage of time immobile, and the mean number of body and arena ipsilesional (left) rotations. To minimize stress, the room was lit as dimly as possible while allowing us to clearly discern the rats. At the beginning of the session, the rat was placed on the right side of the field, with its head facing the wall. A first acquisition was performed the day before the lesion, serving as a reference value, and then acquisitions were performed on days (D) 1, 2, 3, 7, 10, 14, 21, and 30 post-lesion.

## 5. Conclusions

Very few works in the literature concern the involvement of oxytocin in vestibular physiopathology. From a pharmacological point of view, the 1 mg/kg dose tested in the present study shows that short-term oxytocin treatment after vestibular lesion impairs the restoration of some postural and locomotor deficits while improving others. It would then be interesting to test other doses in order to determine the most effective non-toxic dose in our model. However, posturo-locomotor deficits represent only one component of the vestibular syndrome. Indeed, the vestibular syndrome is also accompanied by oculomotor, vegetative, and cognitive disorders. These disorders are experienced by the patient as a stressful and anxiety-provoking experience that disrupts vestibular compensation in some patients. Social isolation in rodents can be considered stressful by the animal, and it is important to consider in the present study that UVN rats were placed in individual cages. Since oxytocin facilitates prosocial behavior in rodents [46] it would be interesting to compare the vestibular compensation with animals housed in groups. More broadly, given the beneficial role of oxytocin in the modulation of stress and anxiety [47,48], it would be interesting to test the effect of this neurohormone on the affective-emotional component present in vestibular pathology [49,50], including persistent postural-perceptual dizziness [51,52].

## Data Availability

The data presented in this study are available in http://doi.org/10.3390/ijms232315262.
